# Cleft Palate Syndrome in the Endangered Spectacled Flying Fox (*Pteropus conspicillatus*): Implications for Conservation and Comparative Research

**DOI:** 10.3390/vetsci10010038

**Published:** 2023-01-05

**Authors:** Lee McMichael, Jennefer Mclean, Jim Taylor, Yissu Martinez, Joanne Meers

**Affiliations:** 1 School of Veterinary Science, University of Queensland, Gatton, QLD 4343, Australia; 2 Tolga Bat Rescue and Research Inc., Carrington Road, Atherton, QLD 4883, Australia; 3 College of Public Health, Medical and Veterinary Sciences, James Cook University, Townsville, QLD 4810, Australia

**Keywords:** cleft palate syndrome, spectacled flying fox, endangered, biodiversity conservation, comparative medicine

## Abstract

**Simple Summary:**

The spectacled flying fox (*Pteropus conspicillatus*) is an endangered frugivorous megabat with a restricted habitat in the Wet Tropics World Heritage Area in far north Queensland, Australia. Its population is in decline, and as such, mortality events in the species require investigation and the development of mitigation strategies. Cleft palate syndrome, first observed in the spectacled flying fox population in 1998, has produced sporadic neonatal mortality events over the past two decades. This study presents the rudimentary morphological signs of cleft palate syndrome in spectacled flying fox neonates, presenting gross pathology of syndromic signs upon visual inspection and a histological examination of palate malformations. It examines syndrome incidence data from the past two decades and reviews recent flying fox research to develop hypotheses related to the causes of the syndrome and to develop future research focuses. This, and future studies, will provide a greater understanding of the risk factors associated with the syndrome to guide the development of mitigation strategies that can deliver conservation outcomes for an endangered species, while presenting a unique opportunity for a novel comparative study into syndromic cleft palate in mammalian species.

**Abstract:**

Cleft palate syndrome, first observed in the spectacled flying fox population in 1998, has produced sporadic neonatal mortality events over the past two decades, with an estimated incidence of up to 1/1000 births per year. This study presents a rudimentary characterisation of the syndrome, presenting gross pathology of syndromic signs upon visual inspection, a histological examination of palate malformations, and syndrome incidence data representing the past two decades. The syndrome presents with a range of signs, primarily congenital palate malformations ranging from a pinhole cleft to a complete hard and soft palate deficit, resulting in the death or abandonment of neonates shortly after birth. The congenital palate malformations are often associated with claw deformities, wiry facial hair, and in some instances, muscle weakness and neurological signs. The natural occurrence of the lethal congenital orofacial birth defects in the spectacled flying fox presents a unique opportunity for the investigation of putative aetiologies, drawing parallels between bat and other mammalian cleft palate risk factors. Further syndrome investigation has the potential to deliver both biodiversity conservation and comparative veterinary and biomedical outcomes.

## 1. Introduction

Orofacial clefts are among the most prevalent human birth defects [1], with an incidence of between 1/1000 to 2.67/1000 of live births in different parts of the world [2]. Cleft lip and cleft palate are variations of a type of congenital deformity caused by abnormal facial development during gestation. The condition is referred to as syndromic when palate malformations appear with other congenital abnormalities in recognizable patterns, or non-syndromic if it appears as an isolated defect [3]. The deformities are not exclusive to the human population, with reports in domestic animal species, inclusive of dogs, cats, horses, cattle, sheep, and goats [4], and sporadic reports in wildlife species. While surgical treatments of cranio-facial birth deformities in humans and companion animals are able to resolve the physical defect, animals with orofacial clefts living in the wild have limited prospects of survival [5].

Congenital palate malformations were first observed in the endangered spectacled flying fox population in flying fox roost sites on the Atherton Tablelands, far north Queensland, Australia, in 1998 [6]. The syndrome appears to exclusively affect the spectacled flying fox, one of four mainland Australian species of flying foxes, and indeed is the only report of megabat palate malformations globally. The natural occurrence of the lethal congenital orofacial birth defects in the spectacled flying fox (*Pteropus conspicillatus*), analogous to syndromic cleft palate in humans, presents a unique opportunity for a novel comparative study into mammalian cleft palate aetiologies. Comparative studies of cleft palate in mammalian species allow the exploration of the relative risk imparted by cleft palate candidate genes and gene–environment interactions. The identification of cleft palate candidate genes has traditionally relied on gene expression and developmental analyses performed in model organisms, particularly the mouse, to identify candidate genes and provide biological plausibility for the association [7]. While examining cleft palate aetiologies in bats is unique, the use of bat models in comparative medicine has precedence in many fields, including reproductive physiology, immunology, and neurobiology [8,9,10].

A number of genes associated with orofacial congenital birth deformities in humans have been identified; however, concordance rates in identical twins—as low as 25 to 45% with notable variability in gene expression and penetrance—support the assumptions that complex environmental triggers also play a role in the development of orofacial deformities [7,11]. There is also an increasing body of evidence that suggests that epigenetic processes contribute to the aetiology of orofacial clefts [12,13,14], whereby inherited changes in gene expression profiles are caused by processes, such as DNA methylation, due to environmental teratogens, rather than changes to the DNA sequence itself [15].

Environmental factors that may interact with a genetic predisposition to orofacial clefts and syndromes, that are most plausibly applicable to human and animal populations, are maternal stress, pathogen infection, and environmental toxin exposure during early gestation. Associations between maternal corticosteroid use in pregnancy and incidence of cleft palate have been reported [16]. Thus, it may be extrapolated that elevations in circulating maternal glucocorticoids, due to stress in early gestation, may contribute to the formation of congenital birth deformities. Natural plant toxins, such as cyanogenic glycosides and teratogenic alkaloids, natural mycotoxins, horticultural fungicides, and heavy metal pollutants are considered to have a causal association with cleft palate [17,18,19]. Pathogenic infections during gestation can increase the risk of foetal malformation, with evidence supporting the association of viral infection with foetal and postnatal morbidity and mortality [20]. As bats are known to host many and varied viral infections [21], it is important to consider in utero viral infection as a putative aetiology of cleft palate in bat species. There is commonality in virus families that have been reported in the aetiology of cleft palate in both human and animal species inclusive of the *Herpesviridae* and *Flaviviridae* families [22,23,24,25,26].

This paper presents a rudimentary characterisation of the morphological signs of cleft palate syndrome in spectacled flying fox neonates, examines the incidence of the syndrome, and reviews current flying fox research to develop hypotheses related to the aetiology of the syndrome and develop future research focus. The natural occurrence of the lethal congenital orofacial birth defects in the spectacled flying fox, analogous to syndromic cleft palate in humans, presents a unique opportunity for a novel comparative study into mammalian cleft palate aetiologies. As such, this paper will also discuss the potential parallels that may be drawn between cross-species risk factors, and how future research may deliver both biodiversity conservation and comparative medicine outcomes.

## 2. Materials and Methods

### 2.1. Animal Ethics and Data Collection

Cleft-palate-syndrome-affected and non-affected spectacled flying fox neonates, and the associated morphological data were collected by volunteers at the Tolga Bat Rescue and Research Inc., Queensland, Australia, under the Rehabilitation Permit WIRP17000616, granted under Nature Conservation (Administration) Regulation 2006. Necropsies of deceased neonates were performed under the Queensland government scientific purposes permit WA0019151 and the University of Queensland animal ethics permit SVS/ANRFA/238/19.

### 2.2. Defect Morphology, Cranial Measurements, and Histology

Deceased neonates were transported frozen to the James Cook University veterinary laboratories for necropsy and histological analysis. Photographs were taken of individual animals showing differing severity of palate malformations and syndromic signs. To compare the cranial development of cleft-palate-syndrome-affected and non-affected neonates, forearm (mm) and head measurements (length from nose to back of head (mm), and width from ear to ear (mm)), were taken from deceased cleft-palate-syndrome-affected neonates (*n* = 49), deceased non-affected neonates (*n* = 21) and live non-affected spectacled flying fox pups (*n* = 34). Cranial and forearm measurements, and severity of syndromic signs, were collected from spectacled flying fox neonates and pups from the years 2011, 2014, 2015, 2018, and 2019. Forearm length is used as a robust estimation of age [27] and was employed as a normalising factor to correct data for age of the neonate/pup. Complete heads were removed from cleft-palate-syndrome-affected and non-affected pups and fixed in formalin. Specimens were decalcified, embedded in paraffin blocks, and sectioned vertically. Histological sections were stained with hematoxylin and eosin stain, examined at 20× magnification, and photographed.

### 2.3. Cleft Palate Incidence

Incidence of cleft palate syndrome in neonatal spectacled flying foxes on the Atherton Tablelands was recorded spanning the years from 2000 to 2019. The data were inclusive of date of orphaning, sex, forearm, and weight measurements. Incidence rates of the syndrome per live births annually were estimated as a ratio of the total predicted births of spectacled flying foxes on the Atherton Tablelands, based on Atherton Tableland spectacled flying fox population counts from 2000 to 2010 [28], under the assumption that 50% of the population are females, and the assertion that on average, 89% of females aged between 3 and 7 years of age produce a pup each year [29].

## 3. Results

### 3.1. Defect Morphology and Histology

**Figure 1:**
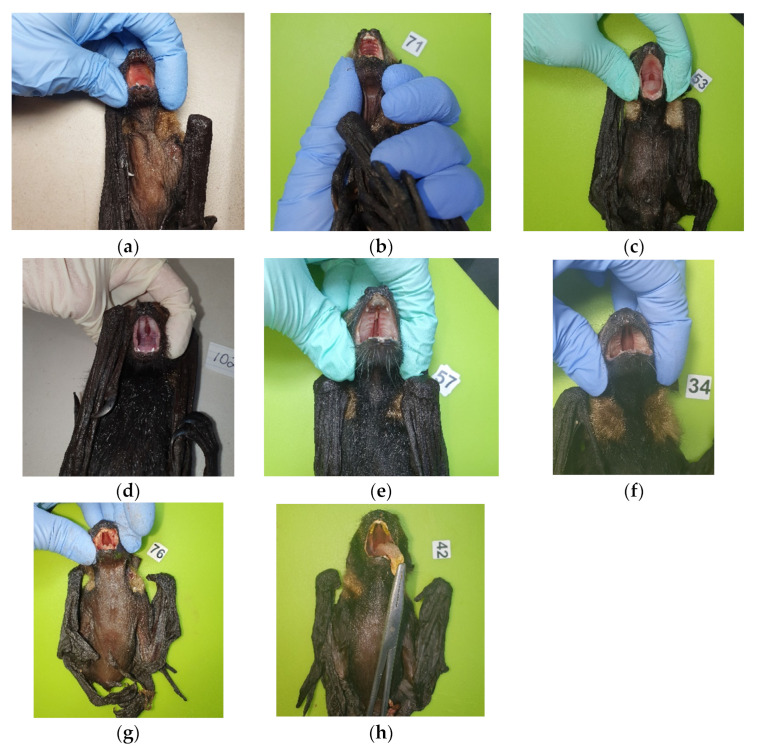
Palate deformities in spectacled flying fox neonates. Normal palate (**a**); minor rostral (anterior) pinhole hard palate cleft (**b**); minor caudal (posterior) soft palate cleft (**c**); midline hard palate fissures (**d**–**f**); complete hard and soft palate deficits (**g**,**h**).

**Figure 2:**
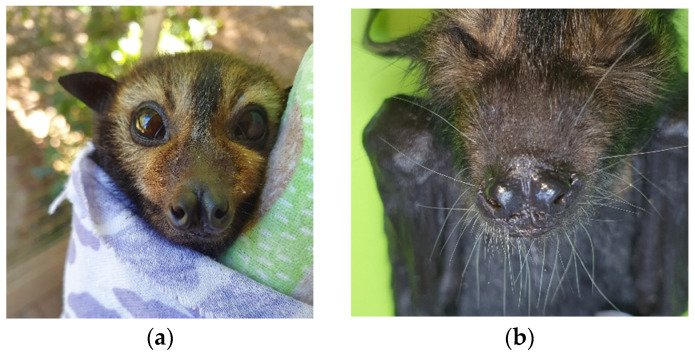

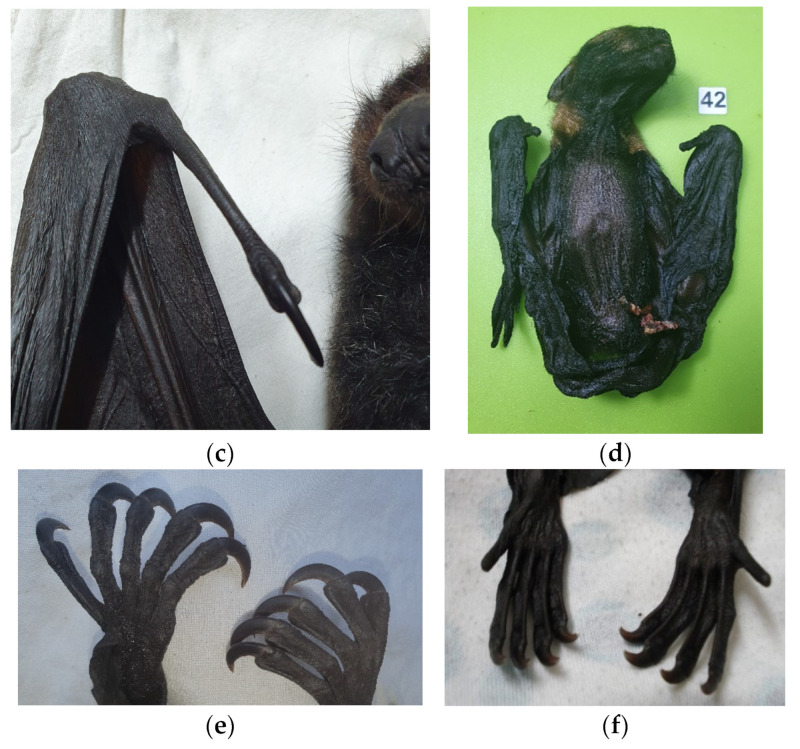
Whisker, nare and claw deformities in spectacled flying fox neonates. Normal nares and whiskers (**a**); wiry whiskers and abnormal development of nares (**b**); normal thumb claw (**c**); abnormal development of thumb claws, missing thumb claws (**d**); normal hind claws (**e**); abnormal hind claw development, missing toe (**f**).

Many deceased, cleft-palate-syndrome-affected spectacled flying fox neonates found at roost sites had been abandoned days after parturition as evidenced by still-attached placenta and umbilicus. Examination of cleft palate affected neonates revealed that many animals presented with a range of hard and soft palate deficit severities (Figure 1), and a range of signs in addition to the palate malformations, including nare, thumb and feet claw deformities, wiry facial hair, and muscle weakness and neurological signs in some instances (Figure 2).

Upon examination of 27 cleft-palate-affected neonates, palate malformations varied from minor pinhole clefts of either the rostral (anterior) or caudal (posterior) of the palate (*n* = 6, 22%), full unilateral midline clefts (*n* = 12, 45%), to complete hard and soft palate deficit (*n* = 9, 33%). Pinhole clefts ranged between 2 mm and 3 mm in circumference, midline palate clefts ranged in width from 2 mm to 6 mm, while severe clefts where the palate was substantially absent were between 7 mm and 8 mm wide.

**Figure 3:**
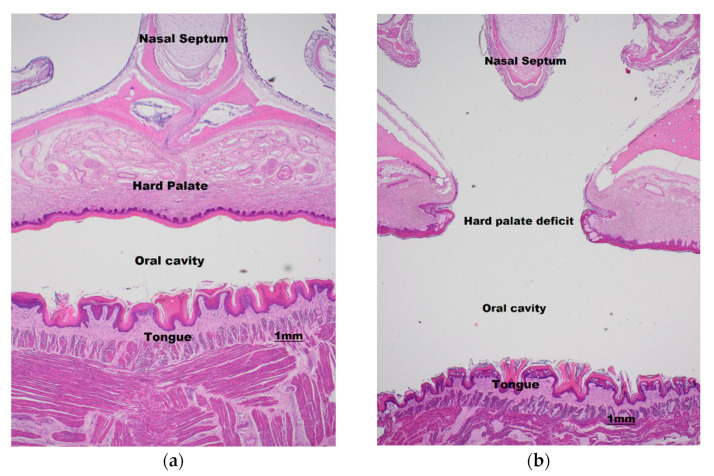
Hematoxylin and eosin-stained 20× maxilla cross section of a normal neonate palate (**a**) and cleft palate syndrome affected neonate (**b**) showing midline hard palate deficit.

In 26% of cases (*n* = 7), cleft palates were combined with other syndromic signs of wiry facial whiskers, thumb, and feet claw abnormalities. Wiry facial whiskers were associated with 5/9 severe cases where palate was substantially absent, 1/10 cases where animals had full midline clefts, and 1/10 cases of minor pinhole clefts. Missing toe or thumb claws were only associated with 2/9 severe cases, both in addition to wiry facial whiskers. Other syndromic signs observed sporadically over the 20-year period included nare deformities, neurological signs, arthrogryposis, cloudy eyes, and leucism. While these reported clinical signs correspond to historical, potentially subjective, and non-controlled observations, the putative association remains of interest. Histological examination suggests the palatal clefts are ectodermal in nature, demonstrating a complete secondary palatal cleft with minimal outgrowth of palatal shelves (Figure 3).

### 3.2. Cranial Measurements

**Figure 4:**
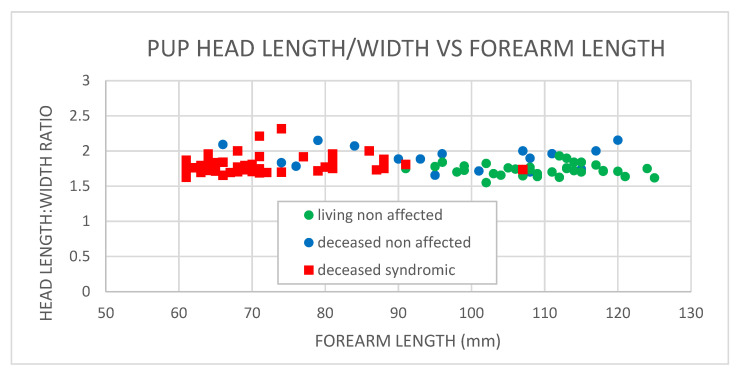
Spectacled flying fox neonate and pup cranial versus forearm measurements. Living non-affected *n* = 34; deceased non-affected *n* = 21; deceased cleft palate syndrome affected *n* = 49.

The comparative cranial measurements from cleft-palate-syndrome-affected and non-affected neonates are presented in Figure 4. A statistical comparison between syndrome-affected (deceased) and non-affected neonates (both living and deceased) demonstrated no statistically significant difference (*p* = 0.528, ANOVA) between the cranial measurements (head length/width). The clustering of deceased neonates to the left of the plot is indicative of the shorter forearm lengths and thus the young age (within days of birth) that the majority of syndrome-affected neonates were found abandoned or deceased. The average respective skull width and length of the cleft-palate-syndrome-affected neonates were 25.2 ± 2.5 mm and 44.6 ± 2.4 mm (*n* = 49), and of the non-affected neonates were 25.8 + 2.8 mm and 48.9 + 4.9 (*n* = 49). The gross morphological and histological data show a congenital syndrome of varying degrees of palate malformations with no association with skull size abnormality.

### 3.3. Cleft Palate Incidence

**Figure 5:**
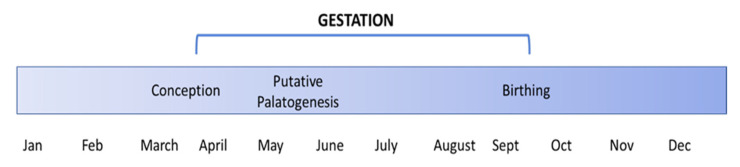
Reproductive timeline of spectacled flying foxes.

*Pteropus conspicillatus* reproduction is synchronous across the population and also with two other Australian flying fox species, *P. alecto* and *P. poliocephalus*. Mating typically occurs in March–April (Australian autumn), followed by 6 months gestation, and birthing beginning in September–October each year (Australian spring). The critical steps in palatogenesis include the growth, alignment, and fusion of the palatal shelves, completed by the end of the first trimester of gestation or prenatal development, in humans (week 9), mice (day 16.5), and canines (day 25–33) [4,30,31]. In the spectacled flying fox, following synchronous mating and conception in March–April each year, the first trimester of pregnancy would coincide with weeks 1–8 of gestation, whereby putative foetal palate formation would occur in June for the majority of pregnancies (Figure 5).

As the majority of cleft-palate-affected neonates are abandoned or die soon after birth, most cleft palate neonates are found early in the birthing season. The palate deficits result in neonates being unable to suckle efficiently, putatively leading to death from starvation or inhalation pneumonia. Other syndromic abnormalities, such as deformed claw and wing development, muscle weakness, and neurological signs [6], most likely lead to the inability of neonates to grasp their mothers, leading to neonatal abandonment. The earliest neonate across the 20-year annual birthing seasons, was found on September 25 and the latest on January 6, with the majority of cleft palate neonates reported in October. The range of dates that the syndrome-affected neonates were recovered may be dependent on the actual timing of conception and the severity of syndromic signs, whereby mild signs may afford a slightly longer longevity. The combined sex ratio across all years of cleft-palate-affected neonates that had sex recorded, was 44% males (*n* = 41) and 56% females (*n* = 53).

**Figure 6:**
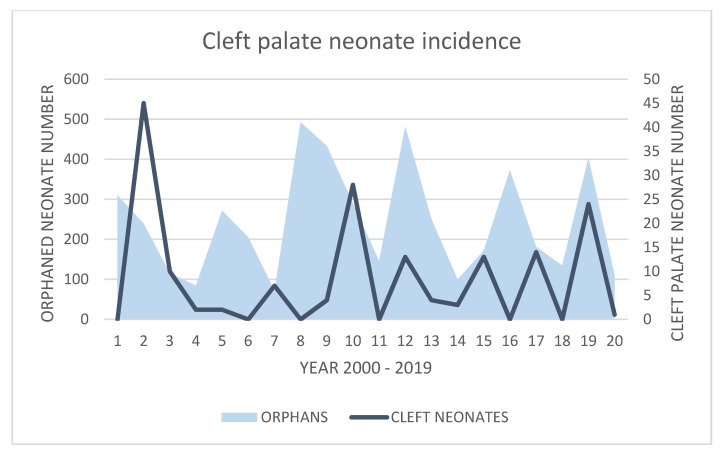
Cleft-palate-syndrome-affected neonates versus total deceased and orphaned neonates by year of birthing season (October–December).

The total annual numbers of cleft-palate-syndrome-affected neonates collected from flying fox roost sites on the Atherton Tablelands annually from 2000 to 2019 is presented in Figure 6. The incidence of the syndrome between the years of 2000 to 2019 varied yearly from nil incidence to an incidence of approximately one syndrome affected neonate per 1000 estimated live births (in 2001). Annual incidences of large numbers of cleft-palate-affected neonates of more than 20 neonates annually occurred only three times in the 20-year period: 45 neonates in 2001, 28 neonates in 2009, and 24 neonates in 2018. Conversely, no cleft-palate-affected neonates were reported in 5 of the 20 years surveyed (2000, 2005, 2007, 2010, 2015, 2017). Of the remaining 12 years over the two decades, 4 years recorded more than 10 cleft palate neonates, and 8 years recorded fewer than 10 neonates. Interestingly, of the three high incidence years, two were preceded by nil incidence years, and one by a very low incidence year of four individuals.

**Figure 7:**
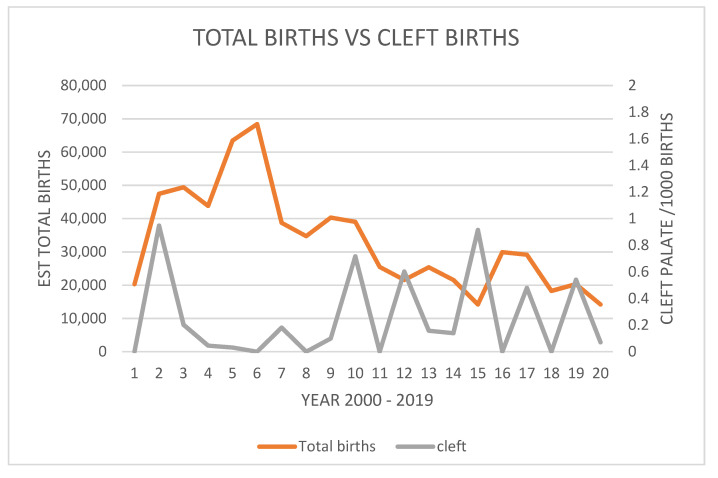
Estimated total births of spectacled flying fox pups versus births of pups with cleft palate syndrome.

The most important finding is that the incidence is sporadic across the 20 years surveyed. Figure 7 demonstrates the estimated live births in the declining population versus the number of cleft-palate-syndrome-affected neonates. Again, this presentation clearly shows a sporadic incidence rather than a uniform incidence, and a putative increasing incidence in some years as the population and estimated live births decline in the latter 10 years of surveyed data.

## 4. Discussion

This study is the first to present a rudimentary characterisation of the cleft palate syndrome in the endangered spectacled flying fox (*Pteropus conspicillatus*), and to discuss parallels between other mammalian cleft palate syndrome aetiologies. The work has potential beneficial biodiversity conservation and comparative biomedical and veterinary outcomes. The spectacled flying fox population is in decline, putatively as a result of decreasing habitat, climate extremes, anthropogenic activities, and more recently, mass mortality events [32,33]. As such, the species was listed in 2019 as endangered in Australia, under the Commonwealth Environment Protection and Biodiversity and Conservation Act 1999. As this species has a low reproductive rate [29], a stable population can only be maintained if the population experiences low year-to-year mortality. Thus, conservation efforts directed towards preventing the decline of this species should focus on reducing mortality rates. It is important that all mortality events be examined when attempting to mitigate the population decline of an endangered species, particularly a species that provides vital ecosystem services to a world heritage ecosystem.

The congenital syndromic malformations observed in the spectacled flying fox are consistent with those in other species. In canines, cleft palate may occur separately or in combination with other developmental abnormalities, with about 8% of cleft-palate-affected canines also affected by birth defects, such as rear limb malformation, hydrocephalus, defects of the heart, or microphthalmia [4,34]. As this study examined only the gross defect morphology upon visual inspection and histopathology of the palate, head, and limbs, in future, more comprehensive post-mortem examinations and computed tomography assessments would be advantageous to determine the precise defect morphology to investigate the entire spectrum of possible phenotypic variation associated with the cleft palate syndrome in the spectacled flying fox. The estimated incidence of cleft palate syndrome of up to 1/1000 live births in spectacled flying fox neonates is consistent with that found in humans [2]. In order to postulate hypotheses around syndrome aetiology, the range of syndrome signs, incidence data, and spectacled flying fox behaviour, particularly during the first trimester of gestation when foetal palate formation is occurring, need to be considered.

Studies exploring the relative risk imparted by cleft palate candidate genes are popular approaches to examining cleft palate aetiology. Genome-wide sequencing and genotyping technologies are becoming more readily available and cost effective, yielding an ever-increasing amount of candidate gene sequence data for many species. These data allow cleft-palate-associated mutations and functional correlations to be identified in human and other animal species which may prove useful in identifying cleft palate candidate genes in the spectacled flying fox. While studies have been published investigating the spectacled flying fox population genetic structure and gene flow between colonies in the Queensland Wet Tropics [35,36], little research has been conducted into disease-causing genetic mutations. With the knowledge that the spectacled flying fox population is in decline, it is possible there has been an increase in defective genes or gene pathway prevalence over time, providing exceptional power to identify defective genetic variants associated with cleft palate syndrome.

If a genetic aetiology is hypothesised, the variability in syndrome signs may indicate that the gene or genes associated with the syndrome display incomplete penetrance, or that there is an interaction between a number of genes to produce a range of signs, or that the syndrome is multifactorial, involving a number of genes and an environmental effect. The gender prevalence of the syndrome, showing an almost equal male and female incidence, does not indicate a clear sex-chromosome-linked syndrome as seen in some human cleft palate genetic studies [37]. Interestingly, syndromic signs do have a similarity to the autosomal dominant human genetic defect associated with Ectrodactyly-ectodermal dysplasia-clefting syndrome, which has syndromic signs inclusive of cleft palate, sparse wiry hair, and distal limb defects inclusive of syndactyly, camptodactyly, and ectrodactyly [38].

The sporadic profile of cleft palate incidence data over a 20-year period is not what would be immediately expected if a genetic disorder was solely involved, whereby a more static incidence year-to-year would be expected. However, the examination of incidence of the syndrome does support a putative increasing incidence in a declining population which may be experiencing a genetic bottleneck in the later 10 years of the data presented. The years of high incidence with intervening years of low incidence supports the hypotheses surrounding a complex gene–environment multifactorial and interactive aetiology of cleft palate syndrome. There is also an increasing body of evidence that suggests that epigenetic processes contribute to the aetiology of orofacial clefts [12,13,14], whereby inherited changes in gene expression profiles are caused by processes, such as DNA methylation due to environmental teratogens, rather than changes to the DNA sequence itself [15].

The most plausible environmental risk factors that may play a key role in cleft palate candidate gene—environment interactions, can be drawn from human studies and recent investigations into spectacled flying fox ecology and behaviour. While we can exclude environmental risk factors such as cigarette smoking, known in humans to be associated with cleft palate development [39], it is plausible to examine environmental factors such as stress, toxin exposure, and pathogen infection. It must be considered, however, that the marked seasonal fluctuations recorded in spectacled flying fox populations in far north Queensland roost sites [40] may confound investigations of putative environmental associations. Seasonal changes in roost site use are thought to be driven by adult animals moving out of roost sites after mating and during the winter gestational months and moving back into roost sites for the warmer birthing season [40]. While this may complicate attempts to pinpoint any location effect for the syndrome during the winter gestational months coinciding with palatogenesis, population studies to address some of the putative environmental interactions have partly been examined.

The hypothetical aetiology of cleft palate syndrome revolving around gestational stressors is complex. While physiological stress impacts on the spectacled flying fox are an important research priority, the adverse effects of stress on foetal development remain contentious. Scialli [39], asserts that most efforts to induce birth defects by restraint and noise stress during pregnancy in rats and mice have yielded negative results. However, in some studies of rats and mice, maternal restraint or food deprivation has produced an increased incidence of cleft palate, an effect possibly mediated by elevated corticosteroids [41,42].

It has been hypothesised that one of the greatest stressors coinciding with winter gestation for spectacled flying foxes may be associated with anthropogenic roost disturbance, namely roost dispersal and construction in urban areas. The stressor effect of flying fox camp anthropogenic dispersal studied by Edson [43], found no direct correlation between elevated urinary cortisol in anthropogenically stressed roost sites, greater than that of cortisol elevations associated with normal life cycle events, such as mating. Further to this work, McMichael [44] compared the population cortisol measurements over three years from far north Queensland spectacled flying fox roost sites, with roost sites in southeast Queensland where black and grey headed flying foxes roosted. Population cortisol measurements from both of the geographically disparate regions demonstrated a synchronous peak in urinary cortisol during the mating season in March/April. However, only the southeast Queensland population demonstrated elevated urinary cortisol levels during winter, putatively due to the physiological stress of thermoregulation in the more southerly cooler climate. Conversely, no elevation in urinary cortisol was recorded in far north Queensland roosts (predominantly spectacled flying foxes) during winter gestation, coinciding with putative embryotic palate formation. Importantly, no incidence of cleft palate syndrome was recorded in flying foxes in southeast Queensland roosts during the study, and thus we conclude that there is no strong evidence to support a correlation between maternal gestational stress and foetal development of cleft palate.

While rainforest fruits have been thought to be the primary food source for spectacled flying foxes [45], stable isotope analysis suggests that non-rainforest flower resources contribute approximately 70% of the metabolized food resources [46], with sclerophyll vegetation providing approximately 45% of metabolized resources and mangroves and orchard/urban areas providing approximately 10% each. This knowledge supports the plausibility of the hypothesis surrounding toxin exposure from both weed species and crop pesticides. The most plausible environmental risk factors to examine include exposure to fungicides employed on horticultural crops, heavy metal pollutants, teratogenic alkaloids found naturally in plant food resources (for example, solasodine), and mycotoxins found on fruit crops (for example, aflatoxins). Naturally occurring toxins are particularly interesting environmental risk factors, as exposure may be governed by fluctuations in climactic conditions, which may explain the yearly fluctuations in cleft palate occurrence in a genetically predisposed population.

To address this hypothesis, an assessment of blood biomarkers of spectacled flying foxes was performed in June 2015, on 50 wild-caught, apparently healthy, spectacled flying foxes in far north Queensland [47]. This assessment sought to establish the general health status of adult spectacled flying foxes at the putative time of foetal palate development, in order to diagnose any chronic or acute health conditions which may provide evidence of toxin exposure. Expected clinical biochemistry changes for chronic toxin exposure may include increased hepatic enzymes levels, whereby toxin exposure, plausibly causing liver enzyme derangement during early pregnancy, is known to cause birth defects in mammals [48]. The blood biomarker values for spectacled flying foxes were temporally comparable to the paraphyletic black flying fox population [49] but did show generally higher eosinophil and liver enzyme values.

While these findings are consistent with putative toxin exposure, the higher prevalence of intraerythrocytic protozoal parasites, subsequently characterised as *Hepatocystis* sp. [50], which were present in 56% of spectacled flying foxes compared to only 4% of black flying foxes, is an interesting parallel to the putative aetiologies of human neonatal cleft palate syndrome. Liver damage caused by parasitic infection may putatively alter retinoid metabolism and vitamin A availability, which has been linked to cleft palate susceptibility [51]. Fluctuations in climactic conditions may govern the exposure to vectors of parasitic liver infections, and thus these types of infections are particularly interesting environmental risk factors which may, again, explain the yearly fluctuations in cleft palate incidence.

An alternative plausible hypothesised infectious agent associated with cleft palate syndrome is viral infection. While bats are known to host a diverse number of viruses of livestock and human health significance, little investigation has been conducted into viral pathogens that may have a conservation impact on endangered bat species. In utero virus infection with individual virus species in virus families as diverse as *Herpesviridae*, *Flaviviridae*, *Paramyxoviridae*, *Picornaviridae*, *Bunyaviridae*, and *Retroviridae* [22,23,24,25,26,52,53], have been shown to be associated with congenital birth deformities in both animal species and humans. Additionally, the interaction between the host genome and viral infection is an important relationship to consider when forming etiological hypotheses for a syndrome that may involve a complex genetic–environment interaction. Host gene variants may contribute to an enhanced susceptibility or resistance to viral diseases, through genes encoding virus receptors, receptor-modifying enzymes, and a range of innate and adaptive immunity-related proteins [54].

## 5. Conclusions

As genome-wide sequencing technologies are readily available and becoming more cost effective, the search for cleft palate candidate genes in the spectacled flying fox is a plausible research goal in combination with investigating putative environmental interactions. Cross-sectional studies of the spectacled flying fox population will facilitate further investigation into toxin exposure and physiological stress scenarios, and thus should be a priority for future research endeavours into the aetiology of cleft palate syndrome of the endangered spectacled flying fox. The natural occurrence of the lethal congenital orofacial birth defects in the spectacled flying fox presents unique opportunities for the investigation of putative aetiologies, drawing parallels between bat and other mammalian species to increase the understanding of risk factors associated with syndromic cleft palate. Further syndrome investigation has the potential to deliver both biodiversity conservation outcomes for a threatened species and comparative veterinary and biomedical outputs.

## Data Availability

Not applicable.
